# Visualizing semipermeability of the cell membrane using a pH-responsive ratiometric AIEgen†
†Electronic supplementary information (ESI) available: Materials and methods; ^1^H NMR and HRMS spectra of compounds; photophysical data and imaging data. See DOI: 10.1039/d0sc02097d


**DOI:** 10.1039/d0sc02097d

**Published:** 2020-05-15

**Authors:** Yuan Gu, Zheng Zhao, Guangle Niu, Han Zhang, Yiming Wang, Ryan T. K. Kwok, Jacky W. Y. Lam, Ben Zhong Tang

**Affiliations:** a Department of Chemical and Biological Engineering , Department of Chemistry , The Hong Kong Branch of Chinese National Engineering Research Center for Tissue Restoration and Reconstruction , Institute for Advanced Study , The Hong Kong University of Science and Technology , Clear Water Bay , Kowloon , Hong Kong 999077 , China . Email: tangbenz@ust.hk; b HKUST-Shenzhen Research Institute , No. 9 Yuexing 1st RD, South Area Hi-tech Park , Nanshan , Shenzhen 518057 , China; c Center for Aggregation-Induced Emission , SCUT-HKUST Joint Research Institute , State Key Laboratory of Luminescent Materials and Devices , South China University of Technology , Guangzhou 510640 , China

## Abstract

In clinical chemotherapy, some basic drugs cannot enter the hydrophobic cell membrane because of ionization in the acidic tumor microenvironment, a phenomenon known as ion trapping. In this study, we developed a method to visualize this ion trapping phenomenon by utilizing a pH-responsive ratiometric AIEgen, dihydro berberine (**dhBBR**). By observing the intracellular fluorescence of **dhBBR**, we found that non-ionized **dhBBR** can enter cells more easily than ionized forms, which is in accordance with the concept of ion trapping. In addition, **dhBBR** shows superior anti-photobleaching ability to *Curcumin* thanks to its AIE properties. These results suggest that **dhBBR** can serve as a bioprobe for ion trapping.

## Abstract 2

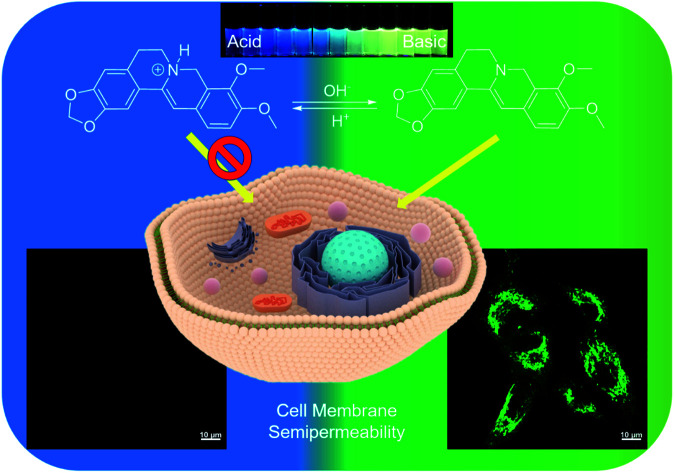
By utilizing a pH-responsive ratiometric AIEgen, dihydro berberine (dhBBR), ion trapping phenomenon was successfully visualized.

## Introduction

All living cells must exchange materials with their extracellular environment in order to be alive.^1^,^2^ The processes of substances entering and exiting cells are controlled by the cell membrane, a biological membrane consisting of a lipid bilayer with proteins embedded in, which separates the interior of cells from their external environment.^3^,^4^ Due to the semipermeable nature of the lipid bilayer, the cell membrane is permeable to non-ionized (fat-soluble) molecules, while the permeability to ionized (water-soluble) molecules is very limited, a phenomenon commonly known as ion trapping.^5^ Partial failure in cancer chemotherapy of some basic drugs, such as vinca alkaloids and anthracyclines, can be ascribed to ion trapping. The acidic tumor microenvironment prevents the ionized alkaline drugs from accumulating in cancer cells.^5^–^9^ Given this, the study of cell membrane's semipermeability, especially ion trapping, is not only fundamentally interesting, but also valuable in improving the clinical chemotherapeutic efficacy.

Ion trapping is most widely studied by HPLC, a well-developed technology.^5^,^9^–^12^ Although HPLC enjoys the advantages of high sensitivity and selectivity, it usually brings about thorny issues like high equipment cost, complexity, complicated sample processing, and long runtime. More worse, the biological sample usually needs to be isolated and homogenized, which makes *in situ* monitoring of biological processes impossible.^13^,^14^

Fluorescence technology, with its charming merits of simplicity, high sensitivity, and low background noise, is becoming more and more popular in biomedical research.^15^ Thanks to the enthusiastic endeavors made by scientists, a lot of fluorescent bioprobes have been developed for various applications.^16^–^19^ Drugs with inherent fluorescence make real-time *in situ* tracking of drug molecules *in vivo* or *in vitro* possible, which is of critical importance in pharmaceutical research. However, so far, fluorescent drugs as probes for monitoring ion trapping have scarcely been reported in spite of their significance in studying drug delivery and drug distribution in the body. This is partly because it is still difficult to find a drug which shows pH-responsive fluorescence. And it is even more challenging to find a drug which has different emission behaviors between its non-ionized and ionized forms. What's more, fluorescent probes usually suffer from the aggregation-caused quenching (ACQ) effect.^20^ In 2001, Tang and co-workers discovered aggregation-induced emission (AIE), which is directly opposite to ACQ.^21^ Since then, a variety of AIEgens have been developed for many advanced applications.^22^,^23^ Recently, Tang *et al.* have put forward natural resources as a new source to explore AIEgens.^24^ AIEgens, usually obtained from herbal plants, animals, and other natural resources, have a lot of unique advantages, such as being synthesis-free, environmentally friendly, pharmaceutically active, *etc.*^24^–^26^ Previously, we have reported that berberine chloride, a natural isoquinoline isolated from Chinese herbal plants, is a rotor-free AIEgen.^24^ In this study, dihydro berberine (**dhBBR**) which is converted from berberine chloride by gut microorganisms was found to be an AIEgen with pH sensitivity. More importantly, the highly similar molecular structure of **dhBBR** to **BBR** enables it to work as a suitable drug-like probe for visualizing cell membrane's semipermeability, thanks to its ratiometric fluorescence.

## Results and discussion

### Synthesis and photophysical properties

**Fig. 1:**
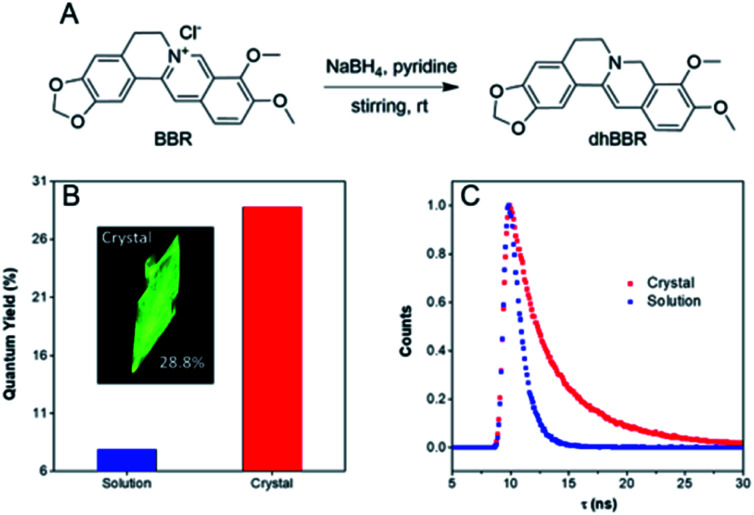
(A) Synthetic route to **dhBBR**. (B) Quantum yield of **dhBBR** in DMSO solution (10 μM) and single crystal states. Excitation wavelength: 365 nm. (C) Time-resolved emission decay curves of **dhBBR** in DMSO solution and single crystal states. Solution concentration: 10 μM; excitation wavelength: 365 nm.

**dhBBR** was synthesized according to the route shown in Fig. 1A with 86% yield.^27^ The purity of the product was confirmed by ^1^H NMR and high resolution mass spectroscopy (HRMS) (Fig. S1 and S3 in the ESI†). The photoluminescence quantum yields (PLQYs) of **dhBBR** in DMSO solution and crystal states were measured to be 7.9% and 28.8%, respectively (Fig. 1B). The results indicate that **dhBBR** is a strong solid-state emitter. The fluorescence lifetime of **dhBBR** in the crystal state (4.68 ns) is longer than that in the solution state (1.1 ns) (Fig. 1C). In addition, from the solution to crystal state, the non-radiative decay rate decreases about 5.5 times (*K*_nr,soln_ = 8.37 × 10^8^ s^–1^, *K*_nr,crystal_ = 1.52 × 10^8^ s^–1^), which should be responsible for the AIE properties of **dhBBR**.

### Mechanism study

**Fig. 2:**
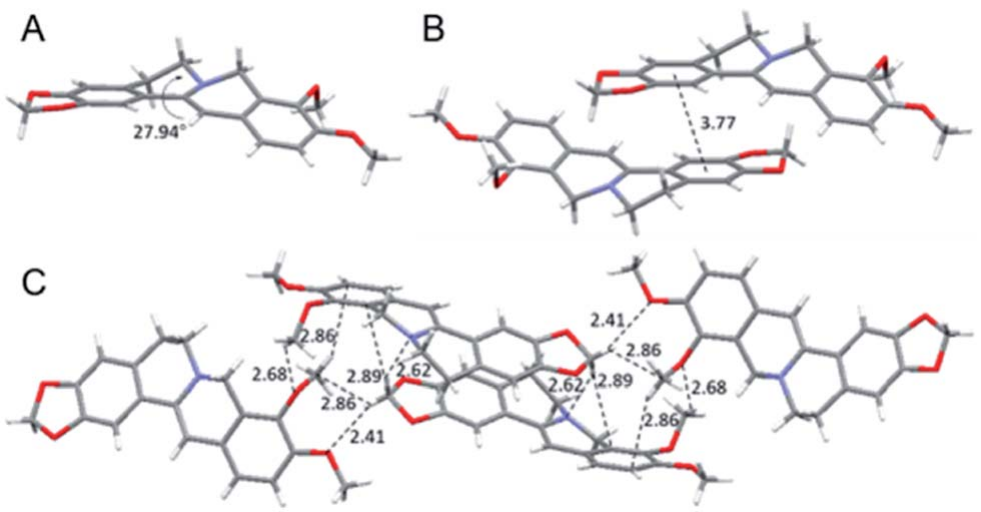
Single crystal packing of **dhBBR**. (A) Intramolecular torsional angle. (B) Intermolecular distance of adjacent molecules. (C) Intermolecular interactions.

To have a clear picture of the photophysical properties of **dhBBR**, the single-crystal structure of **dhBBR** was analyzed.^28^ As shown in Fig. 2, the conformation of **dhBBR** in the crystal state is non-planar with a 27.94° twisted angle, which indicates that intramolecular vibration is possible. In addition, the intermolecular distance of the adjacent **dhBBR** molecule aligned in parallel was measured to be 3.771 Å, which exceeds the effective π–π stacking distance (3.5 Å) to quench the fluorescence.^29^ What's more, multiple intermolecular interactions (2.408–2.888 Å in distance) contribute to rigidification of the molecular conformation which makes **dhBBR** highly emissive in the crystal state. Thus, the brighter emission of **dhBBR** in the crystal state than that in solution can possibly be explained by the restriction of intramolecular vibration which suppresses the non-radiative decay pathway.^30^ To further clarify the intramolecular vibration for determining the photophysical properties of **dhBBR**, viscosity- and temperature-dependent fluorescence changes of **dhBBR** were investigated. As shown in Fig. S4(A and B),† **dhBBR** shows stronger emission in viscous solvents or at low temperature because the intramolecular vibration is suppressed.

### pH-dependent ratiometric fluorescent response

**Fig. 3:**
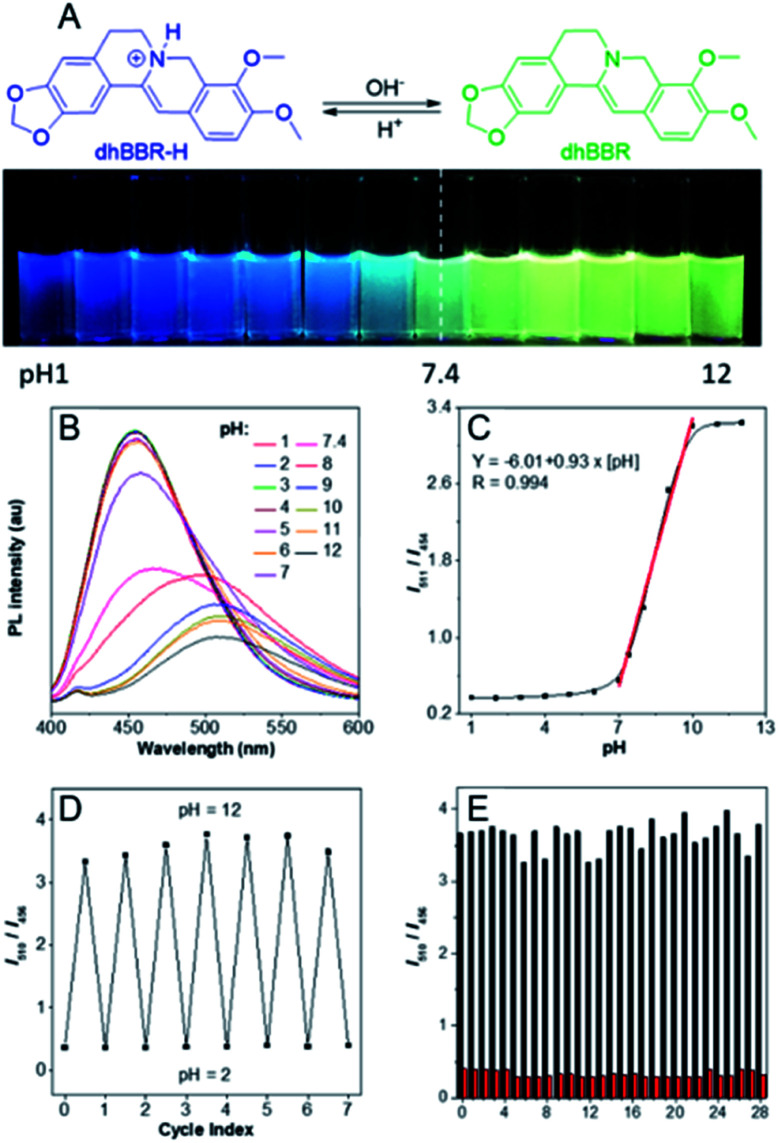
(A) Schematic illustration of **dhBBR**'s fluorescent response to pH change. (B) Emission spectra of **dhBBR** in PBS buffer solutions with different pH values. [**dhBBR**] = 10 μM. (C) Plot of *I*_511_/*I*_454_*versus* pH. *I*_511_ and *I*_454_ denote the emission intensities of the solution at 511 and 454 nm, respectively. Excitation wavelength: 365 nm. (D) Fluorescence reversibility of **dhBBR** in PBS buffer between pH 2.0 and pH 12.0. [**dhBBR**] = 10 μM. (E) Ratiometric fluorescent responses of **dhBBR** (10 μM) to different potential interfering agents in pH 3.0 (red column) and 10.0 (black column) PBS buffer solutions: (0) control; (1) Na^+^; (2) K^+^; (3) Ca^2+^; (4) Mg^2+^; (5) Fe^2+^; (6) Cu^2+^; (7) Zn^2+^; (8) Mn^2+^; (9) Al^3+^; (10) Pb^2+^; (11) Ac^–^; (12) SO_4_^2–^; (13) NO_3_^–^; (14) PO_4_^2–^; (15) S_2_O_3_^2–^; (16) CO_3_^2–^; (17) Phe; (18) His; (19) Met; (20) Pro; (21) Arg; (22) Asp; (23) Cys; (24) Hcy; (25) GSH; (26) glucose; (27) H_2_O_2_; (28) NaClO. Conditions: *λ*_ex_ = 365 nm.

The nitrogen atom in the molecular backbone of **dhBBR** is speculated to be protonated in acid.^31^,^32^ As shown in Fig. 3A, **dhBBR** exhibits obvious pH-dependent fluorescence. Adding acid to **dhBBR** causes a gradual blue shift of **dhBBR**'s emission from 511 nm to 454 nm as shown in Fig. 3A. To understand **dhBBR**'s pH-dependent fluorescence, an absorption titration experiment is performed. As shown in Fig. S5,† there is a red shift of the maximum absorption band of **dhBBR** from 350 nm to 426 nm when the pH of the buffer was changed from 2 to 9. A ^1^H NMR titration study was performed by adding trifluoroacetic acid (TFA) to **dhBBR**. As shown in Fig. S2,† there are downfield shifts of the isoquinoline protons induced by TFA. The new peak at 8.0 suggested that the nitrogen atom in the isoquinoline is protonated. A pH titration experiment was then conducted. The fluorescence maximum of **dhBBR** exhibited a gradual red shift when the pH increased from 1 to 12 (Fig. 3B). More importantly, **dhBBR** showed an excellent linearity of *I*_511_/*I*_454_ in the pH range of 7–10 with a p*K*_a_ value of 8.4 (Fig. 3C), which indicated that **dhBBR** can serve as a ratiometric pH probe. Furthermore, **dhBBR** showed a good reversibility between pH 2 and pH 12 as shown in Fig. 3D. In addition, the intensity ratios (*I*_511_/*I*_454_) of **dhBBR** in pH 3 and pH 10 buffer remain nearly unchanged after 10 min (Fig. S6†). The fluorescence response of **dhBBR** towards different interfering species was evaluated (Fig. 3E). The intensity ratio (*I*_511_/*I*_454_) was negligibly affected by common metal ions (Na^+^, K^+^, Ca^2+^, Mg^2+^, Fe^2+^, Cu^2+^, Zn^2+^, Mn^2+^, Al^3+^, and Pb^2+^; 0.1 mM for Na^+^, K^+^, Ca^2+^, and Mg^2+^ and 0.01 mM for Fe^2+^, Cu^2+^, Zn^2+^, Mn^2+^, Al^3+^, and Pb^2+^), negatively charged species (Ac^–^, SO_4_^2–^, NO_3_^–^, PO_4_^2–^, S_2_O_3_^2–^, and CO_3_^2–^; 0.01 mM for SO_4_^2–^, PO_4_^2–^, S_2_O_3_^2–^, and CO_3_^2–^ and 0.02 mM for Ac^–^ and NO_3_^–^), amino acids (Phe, His, Met, Pro, Arg, Asp, Cys, and Hcy; 0.01 mM for each), GSH (0.01 mM), glucose (0.01 mM), and reactive oxygen species (H_2_O_2_, 0.01 mM; NaClO, 0.01 mM).

### Visualizing semipermeability of the cell membrane

**Fig. 4:**
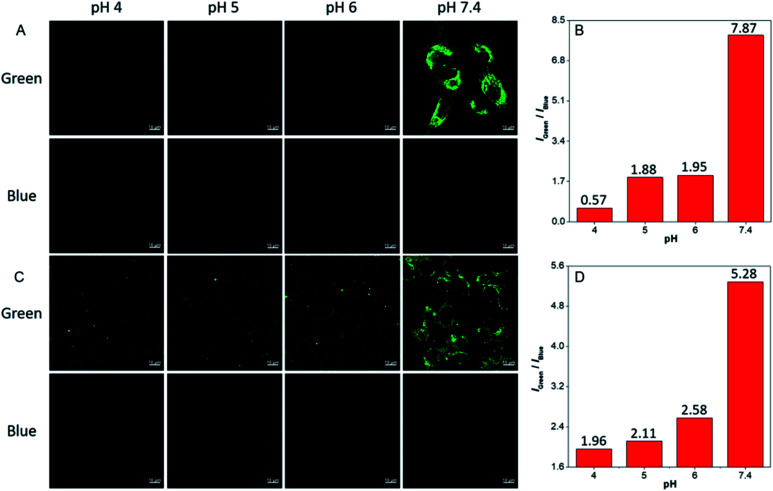
CLSM images of A549 cells (A) and HEK 293T cells (C) stained with **dhBBR** (1 μM) in PBS buffer with different pH for 30 min. (B and D) Relative PL intensity of **dhBBR** treated A549 cells (B) and HEK 293T cells (D). Scale bar = 10 μm.

**Fig. 5:**
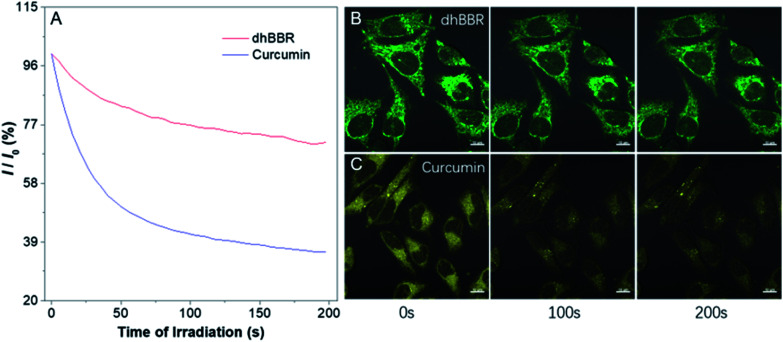
(A) Photostability of **dhBBR** and *Curcumin* under continuous scanning at 405 nm. *I*_0_ is the initial PL intensity, while *I* is that of the corresponding sample after a designated time of irradiation. (B and C) CLSM images of HeLa cells stained with (B) **dhBBR** (1 μM) and (C) *Curcumin* (1 μM) before and after 200 s of light irradiation. All the images share the same scale bar: 10 μm.

The ratiometric fluorescent response of **dhBBR** towards pH and the pharmacological properties of **dhBBR**^33^,^34^ inspired us to further explore the potential of **dhBBR** to be utilized as a probe for ion trapping. Before such an exploration, the biocompatibility of **dhBBR** with various cell lines was investigated first. As shown in Fig. S7,† **dhBBR** shows little toxicity towards different cell types at a concentration of 1 μM (with more than 80% cells being alive), indicating the feasibility of a cell imaging study. For cell imaging, different from the reported pH-responsive probes for which nigericin and monensin are usually used for intracellular pH calibration,^35^–^38^ we didn't use any chemicals to calibrate intracellular pH because we wanted intracellular pH to be constant. Instead, we incubated cells with **dhBBR**-containing PBS buffer of varied pH (pH from 4 to 7.4) for a period. Cells were then imaged under confocal microscopy. It was intriguing to notice that A549 cells incubated with **dhBBR**-containing PBS with a pH range of 4–6 show almost no emission from both blue and green channels (blue channel: 400–460 nm; green channel: 500–600 nm) (Fig. 4A). In contrast, obvious green fluorescence was observed inside cells when A549 cells were incubated with **dhBBR**-containing PBS buffer of pH 7.4 (Fig. 4A). Also, increasing the pH of the PBS buffer from 4 to 7.4 can also induce a gradual increase of the emission ratio (*I*_Green_/*I*_Blue_) (Fig. 4B). Similar results were also observed in HEK 293T cells (Fig. 4C and D). Additionally, different concentrations of **dhBBR** were used and similar results were obtained in HeLa cells (Fig. S8†). This “acid out, base in” phenomenon can be ascribed to the semipermeability of the cell membrane as mentioned above. Due to the hydrophobic nature of the cell membrane, it is easier for non-ionized **dhBBR** to enter cells than for ionized forms, a phenomenon known as “ion trapping”, which well explains why incubating cells with **dhBBR**-containing PBS of higher pH value causes stronger intracellular fluorescence than in the case of **dhBBR**-containing PBS with lower pH value. Photostability is an important parameter in evaluating a bio-probe's anti-photobleaching ability. As shown in Fig. 5, more than 70% of **dhBBR**'s fluorescence is retained even after 200 seconds of irradiation, while less than 40% of *Curcumin*'s fluorescence is retained after the same irradiation time, suggesting that **dhBBR** has superior anti-photobleaching ability compared to *Curcumin* thanks to its AIE properties.

## Conclusions

In summary, dihydro berberine (**dhBBR**) was found to be an AIEgen. The single crystal analysis, viscosity effect, and low-temperature effect revealed that the AIE phenomenon of **dhBBR** originates from the restriction of intramolecular vibration (RIV). Moreover, **dhBBR** can serve as a fluorescent probe for visualizing ion trapping thanks to its ratiometric fluorescent response to pH and superior anti-photobleaching ability.

## Experimental procedures

### Materials and instrumentation

Chemicals for synthesis were purchased from Sigma-Aldrich or Meryer and used as received without any further purification. Dihydro berberine was prepared according to the reported literature.^39^ The ^1^H-NMR spectrum was obtained on a Bruker AV 400 spectrometer. High resolution mass spectra (HRMS) were recorded on a GCT premier CAB048 mass spectrometer operating in MALDI-TOF mode. Ultraviolet-visible (UV-vis) absorption spectra were taken on a PerkinElmer Lambda 25 UV-vis absorption spectrophotometer. Photoluminescence spectra were recorded on a PerkinElmer LS 55 fluorescence spectrometer. The absolute fluorescence quantum yields were measured on a Hamamatsu Absolute Quantum Yield Spectrometer C11347.

### Synthesis of dihydro berberine (**dhBBR**)

Anhydrous berberine hydrochloride (1 mmol, 371.8 mg) and sodium borohydride (1 mmol, 37.8 mg) were dissolved in pyridine, and then the mixture was stirred slowly at room temperature. After that, ice water was added to the system. The obtained light yellow powdery solid was filtered, dried *in vacuo* to afford pure **dhBBR** (290.1 mg, 86%). ^1^H NMR (400 MHz, CDCl_3_, 25 °C), *δ* (ppm): 7.14 (1H), 6.71 (2H), 6.55 (1H), 5.92 (3H), 4.29 (2H), 3.82 (6H), 3.12–3.09 (2H), 2.87–2.84 (2H); HRMS (MALDI-TOF, *m*/*z*): [M]^+^ calcd. for C_20_H_19_NO_4_, 337.1314; found, 337.1320.

### Spectral measurement

The absorption and emission spectra were measured in PBS buffer solutions (10 mM). A stock solution of **dhBBR** (1 mM) was prepared in DMSO and was subsequently diluted to prepare 10 μM solutions of **dhBBR** in PBS buffer with various pH (1, 2, 3, 4, 5, 6, 7, 7.4, 8, 9, 10, 11, and 12). PBS buffer solutions (10 mM) with varied pH were prepared by using NaOH (1.0 M) or HCl (1.0 M) to adjust the pH. For the calibration curve, 20 μL of stock solutions of **dhBBR** (1 mM) were mixed with 980 μL of PBS buffer with different pH in a quartz optical cell with a 1.0 cm optical path length at 25 °C, and spectral data were recorded immediately. Excitation was at 365 nm and emission was detected at 454 nm and 511 nm.

### Cell culturing

HeLa cells, A549 cells, and HEK 293 T cells were purchased from ATCC. All cells were cultured in Dulbecco's Modified Eagle's Medium with 1% penicillin–streptomycin and 10% FBS, at 37 °C in a humidified incubator with 5% CO_2_. The culture medium was replaced every second day. By treating with 0.25% trypsin–EDTA solution, the cells were collected after they reached confluence.

### Cytotoxicity assay

HeLa cells, A549 cells, and HEK 293T cells were seeded in 96-well plates at a density of 5000 cells per well, respectively. After 24 h of cell culture, various concentrations of **dhBBR** were added to the 96-well plates. After another 24 h of cell culture, the medium was removed and freshly prepared MTT medium solution (0.5 mg mL^–1^, 100 μL) was added to the 96-well plates. After incubation at 37 °C, with 5% CO_2_, for 6 h, the MTT medium solution was removed carefully. After that, 100 μL of DMSO was added to each well and the plate was gently shaken at room temperature to dissolve all the formed precipitates. A microplate reader was utilized to measure the absorbance at 570 nm from which the cell viability could be determined. Cell viability was expressed as the ratio of absorbance of the cells incubated with **dhBBR** solution to that of the cells incubated with culture medium only.

### Cell imaging

Cells were grown in a 35 mm Petri dish with a cover slip at 37 °C, with 5% CO_2._ First, cells were incubated with different-pH buffer solutions containing **dhBBR** (0.5, 1, and 10 μM) or *Curcumin* (10 μM) for 30 min at 37 °C, with 5% CO_2_. Then, the staining solution was removed and the cells were washed with PBS of the same pH as the staining solution three times. After that, the cells were imaged using confocal microscopy (Zeiss laser scanning confocal microscope LSM7 DUO). For **dhBBR**, the excitation wavelength was 405 nm, and the emission filter was 400–460 nm and 500–600 nm, respectively; for *Curcumin*, the excitation wavelength was 405 nm, and the emission filter was 500–600 nm.^40^

### Photobleaching assay

HeLa cells stained with **dhBBR** were irradiated using a 405 nm laser for 200 s continuously using confocal microscopy to evaluate **dhBBR**'s photostability. For comparison, HeLa cells stained with *Curcumin* were irradiated using a 405 nm laser under the same conditions.

## Conflicts of interest

There are no conflicts to declare.

## Supplementary Material

Supplementary information
