# Supplementary material for: Visualizing semipermeability of the cell membrane using a pH-responsive ratiometric AIEgen
Source: Chem Sci. 2020 May 15;11(22):5753–8. doi: 10.1039/d0sc02097d (PMC7422962; doi:10.1039/d0sc02097d)
Supplement: Supplementary file 1 [file SC-011-D0SC02097D-s001.pdf]

Electronic Supplementary Material (ESI) for Chemical Science.
This journal is © The Royal Society of Chemistry 2020

Electronic Supplementary Information

Visualizing Semipermeability of Cell Membrane by a pH-responsive Ratiometric AIEgen

Yuan Gu,[ab] Zheng Zhao,[ab] Guangle Niu,[ab] Han Zhang,[c] Yiming Wang,[ab] Ryan T. K. Kwok,[ab] Jacky W. Y. Lam,[ab] and Ben Zhong Tang*[abc]

[a] Department of Chemical and Biological Engineering, Department of Chemistry, the Hong Kong Branch of Chinese National Engineering Research Center for Tissue Restoration and Reconstruction and Institute for Advanced Study, The Hong Kong University of Science and Technology, Clear Water Bay, Kowloon, Hong Kong 999077, China.

[b] HKUST- Shenzhen Research Institute, No. 9 Yuexing 1st RD, South Area Hi-tech Park, Nanshan, Shenzhen 518057 China.

[c] Center for Aggregation-Induced Emission, SCUT-HKUST Joint Research Institute, State Key Laboratory of Luminescent Materials and Devices, South China University of Technology, Guangzhou 510640, China.

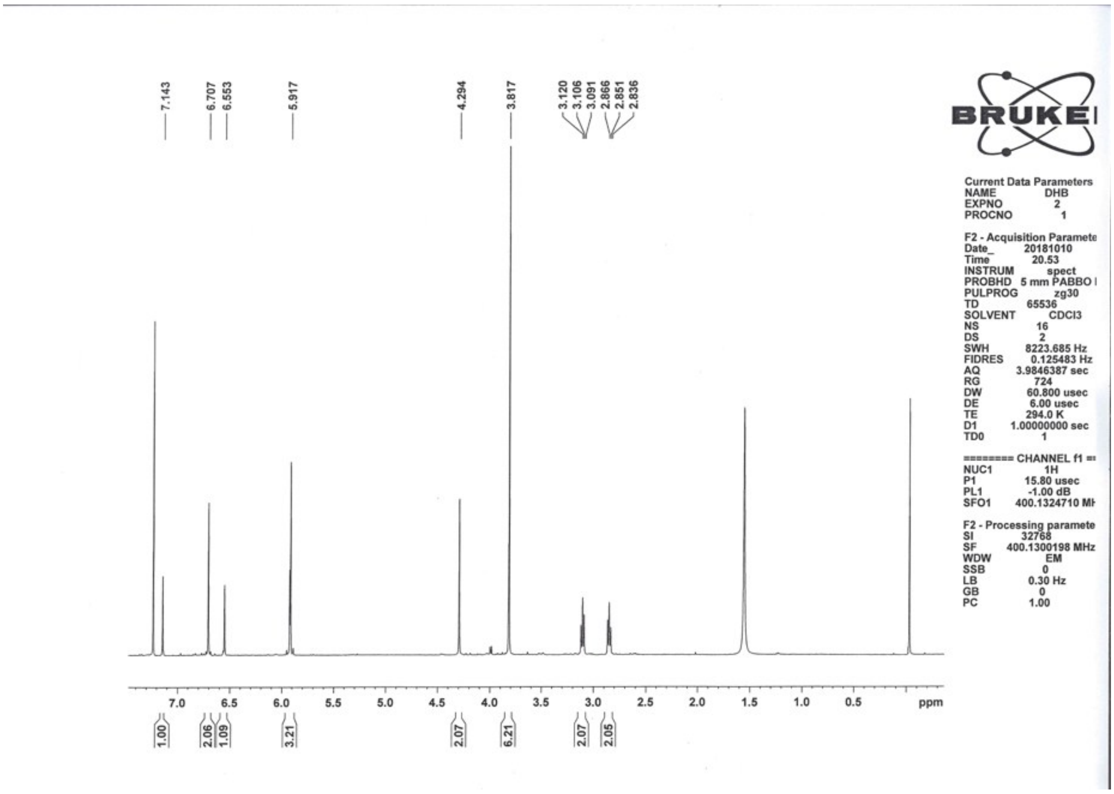

Fig. S1 ^1H NMR spectrum of dhBBR in $CDCl_3$.

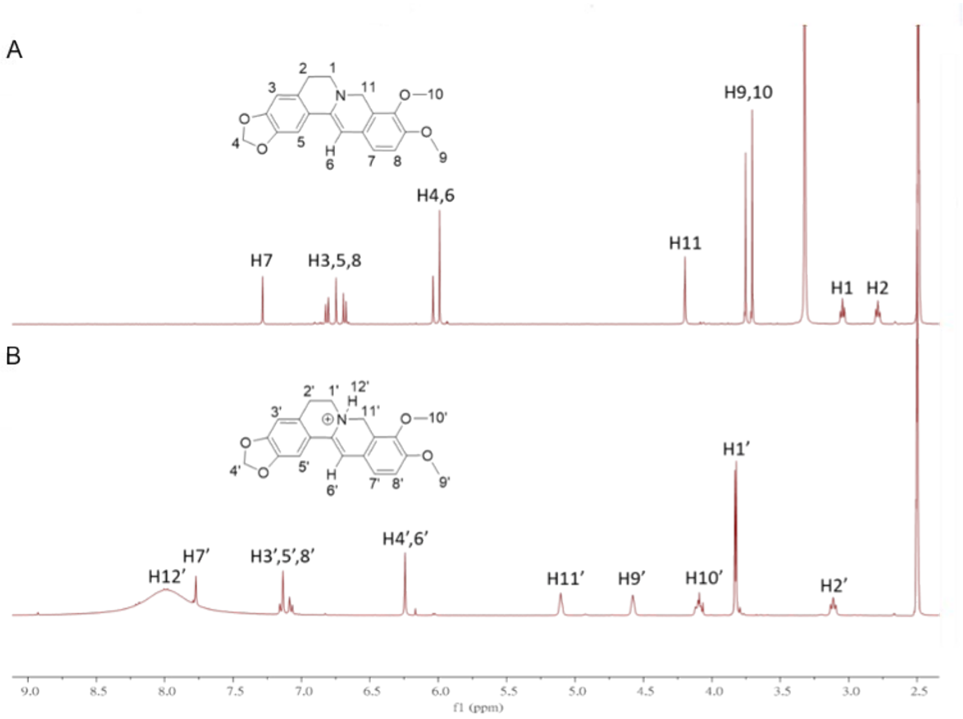

Fig. S2 ^{1}H NMR spectra of dhBBR before (A) and after (B) the addition of trifluoroacetic acid in DMSO-d_6 solution.

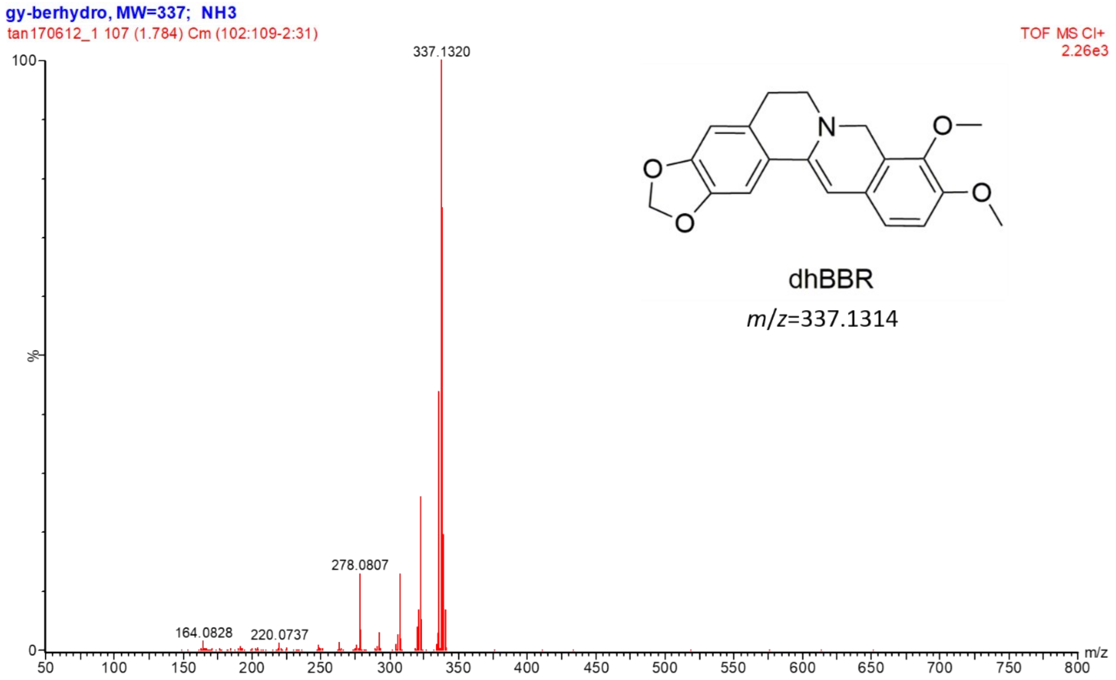

Fig. S3 HRMS spectrum of dhBBR.

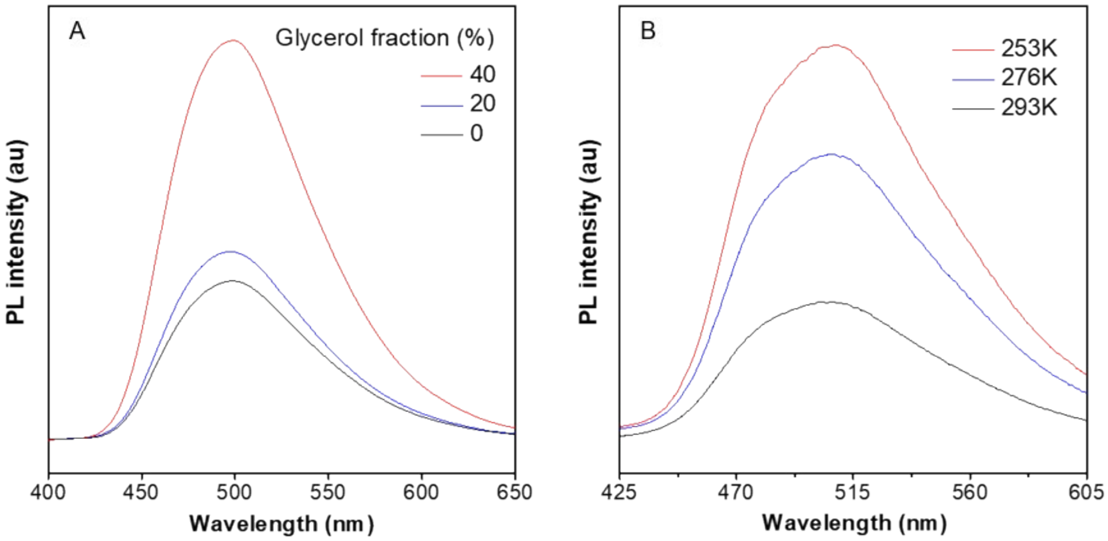

Fig. S4 (A) PL spectra of dhBBR in ethylene glycol/glycerol mixtures with different fractions of glycerol. (B) Temperature dependence of the PL spectra of dhBBR in DMSO solution. Solution concentration: 10 µM; Excitation wavelength: 365 nm.

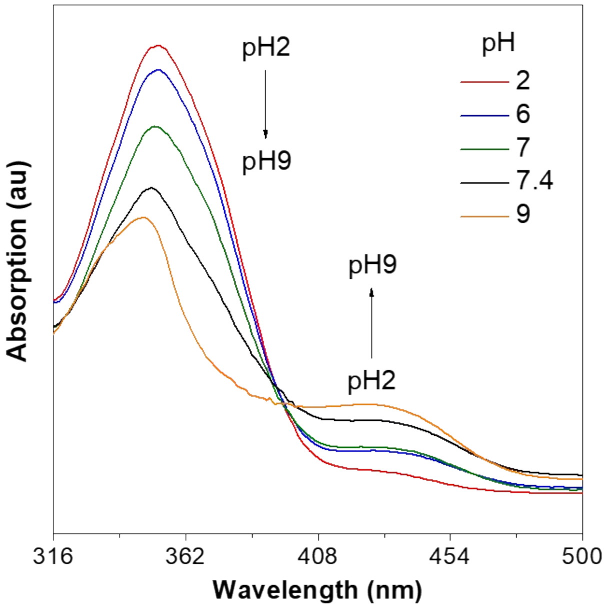

Fig. S5 UV-Vis spectra of dhBBR in the PBS buffer solutions with different pH values. [dhBBR] = 10 μM.

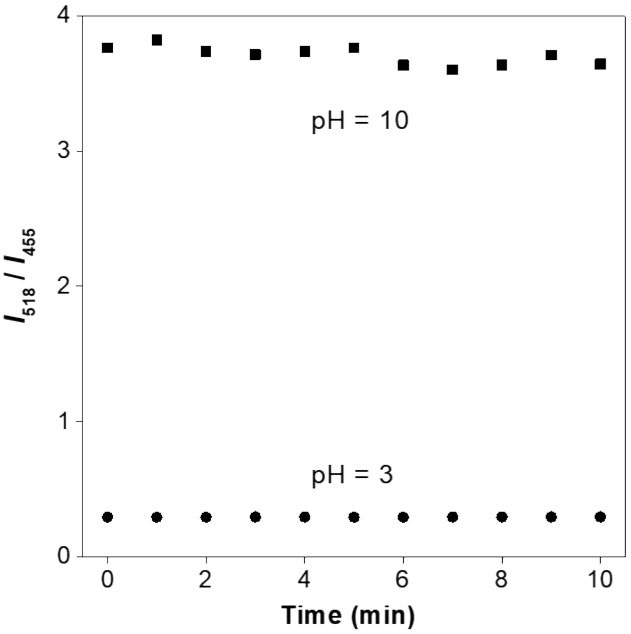

Fig. S6 Time course of the fluorescence intensity ratios (I_{518}/I_{455}) of dhBBR (10 μM) in PBS buffer at pH 3 and pH 10.

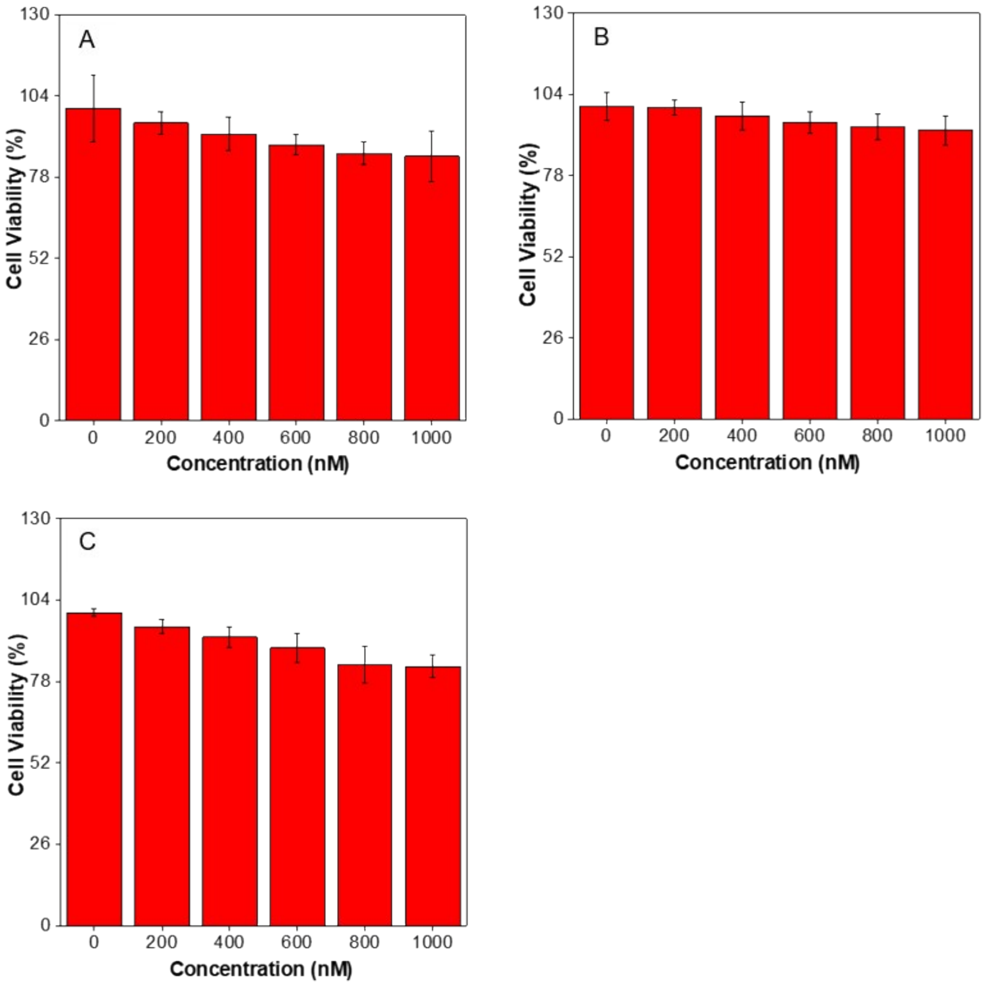

Fig. S7 Viability of A549 cells (A), HEK 293T cells (B) and HeLa cells in the presence of different concentrations of dhBBR for 24 h. Data are expressed as mean value of six separate trials.

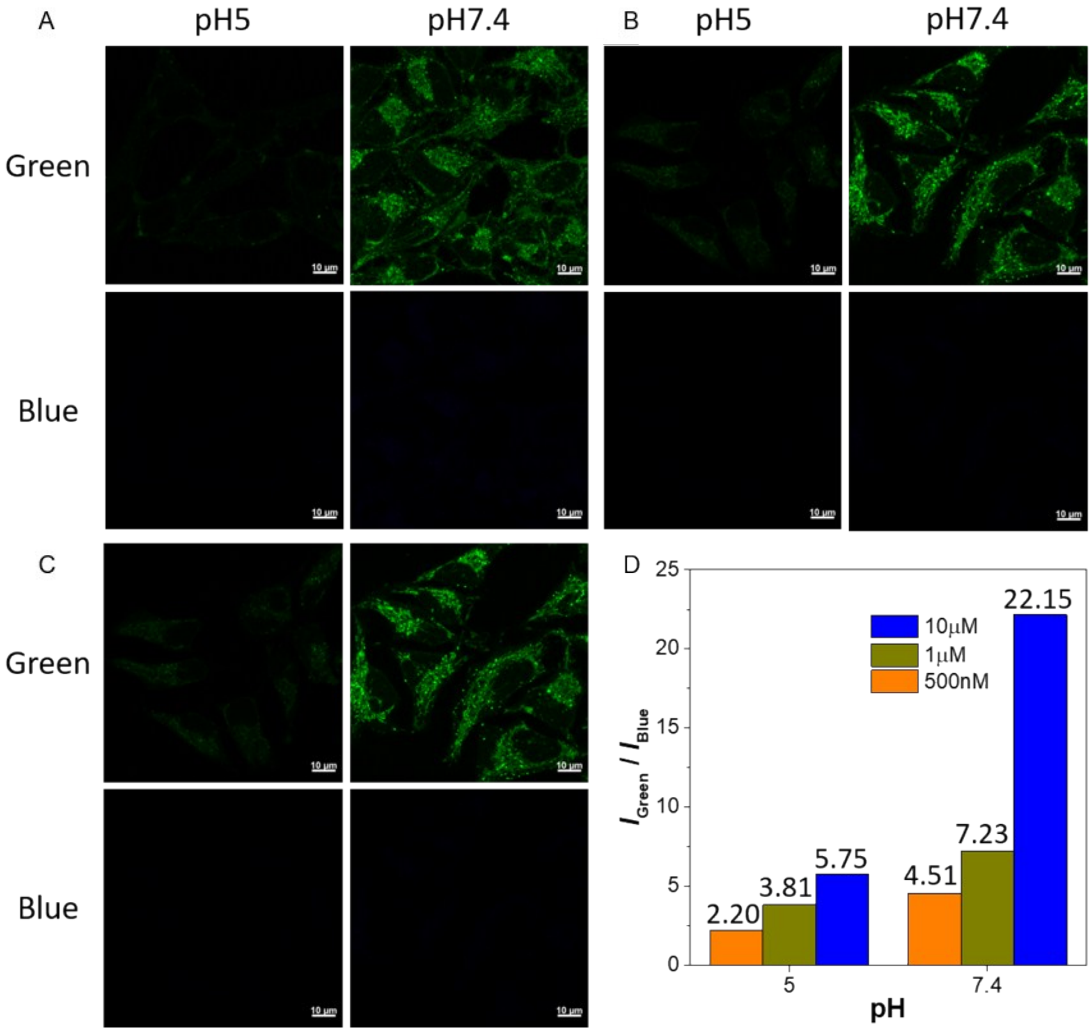

Fig. S8 CLSM images of HeLa cells stained with varied concentrations of dhBBR in different pH PBS buffers for 30 min. dhBBR Concentrations: (A) 500 nM. (B) 1μM. (C) 10μM. (D) Relative PL intensity of varied concentrations of dhBBR treated HeLa cells in different pH PBS buffers. Scale bar = 10 μm.
